# Better Together: Reliable Application of the Post-9/11 and Post-Iraq US Intelligence Tradecraft Standards Requires Collective Analysis

**DOI:** 10.3389/fpsyg.2018.02634

**Published:** 2019-01-07

**Authors:** Alexandru Marcoci, Mark Burgman, Ariel Kruger, Elizabeth Silver, Marissa McBride, Felix Singleton Thorn, Hannah Fraser, Bonnie C. Wintle, Fiona Fidler, Ans Vercammen

**Affiliations:** ^1^Department of Philosophy, University of North Carolina at Chapel Hill, Chapel Hill, NC, United States; ^2^Centre for Philosophy of Natural and Social Science, London School of Economics and Political Science, London, United Kingdom; ^3^Centre for Environmental Policy, Imperial College London, London, United Kingdom; ^4^School of Biosciences, University of Melbourne, Parkville, VIC, Australia; ^5^School of Historical and Philosophical Studies, University of Melbourne, Parkville, VIC, Australia; ^6^Melbourne School of Psychological Sciences, University of Melbourne, Parkville, VIC, Australia; ^7^Centre for the Study of Existential Risk, University of Cambridge, Cambridge, United Kingdom

**Keywords:** intelligence analysis, intelligence failures, intelligence reform, IRTPA, ICD203, ODNI, tradecraft standards, inter-rater reliability

## Abstract

**Background:** The events of 9/11 and the October 2002 National Intelligence Estimate on Iraq’s Continuing Programs for Weapons of Mass Destruction precipitated fundamental changes within the United States Intelligence Community. As part of the reform, analytic tradecraft standards were revised and codified into a policy document – Intelligence Community Directive (ICD) 203 – and an analytic ombudsman was appointed in the newly created Office for the Director of National Intelligence to ensure compliance across the intelligence community. In this paper we investigate the untested assumption that the ICD203 criteria can facilitate reliable evaluations of analytic products.

**Methods:** Fifteen independent raters used a rubric based on the ICD203 criteria to assess the quality of reasoning of 64 analytical reports generated in response to hypothetical intelligence problems. We calculated the intra-class correlation coefficients for single and group-aggregated assessments.

**Results:** Despite general training and rater calibration, the reliability of individual assessments was poor. However, aggregate ratings showed good to excellent reliability.

**Conclusion:** Given that real problems will be more difficult and complex than our hypothetical case studies, we advise that groups of at least three raters are required to obtain reliable quality control procedures for intelligence products. Our study sets limits on assessment reliability and provides a basis for further evaluation of the predictive validity of intelligence reports generated in compliance with the tradecraft standards.

## Introduction

In a seminal article on the role of intelligence analysis, Betts wrote that “the role of intelligence is to extract certainty from uncertainty and to facilitate coherent decision in an incoherent environment” (Betts, 1978, p. 69). In other words, the role of intelligence analysis is to *reduce* (but not necessarily eliminate, see Heazle, 2010; Marrin, 2012) and *caveat* uncertainty (Friedman and Zeckhauser, 2012) to improve national security policy. However, in the wake of perceived intelligence failures such as predicting the 9/11 attacks (National Commission on Terrorist Attacks Upon the United States, 2004, but also see Zegart, 2005, 2006) and Iraq’s capability of deploying weapons of mass destruction (The Commission on the Intelligence Capabilities of the United States Regarding Weapons of Mass Destruction, 2005, but also see Phythian, 2006), the intelligence community’s (IC) ability to help policy makers manage uncertainty was criticized. In consequence, the United States Congress passed sweeping reforms in the 2004 Intelligence Reform and Terrorism Prevention Act (IRTPA), demanding, among other things, the adoption of analytic tradecraft standards to improve the quality of reasoning and argumentation in intelligence products. Moreover IRTPA mandated the creation of an ombudsman for analytic integrity to ensure “finished intelligence products produced […] are timely, objective, independent of political considerations, based upon all sources of available intelligence, and employ the standards of *proper analytic tradecraft*” (IRTPA, 2004, Section 1019a, our emphasis).

In response, the Director of National Intelligence signed Intelligence Community Directive [ICD] 203 (2007/2015), specifying four analytic standards: objectivity, political independence, timeliness, and good tradecraft. The latter further identifies nine elements of analytic tradecraft: (1) Properly describes quality and credibility of underlying sources, data, and methodologies; (2) Properly expresses and explains uncertainties associated with major analytic judgments; (3) Properly distinguishes between underlying intelligence information and analysts’ assumptions and judgments; (4) Incorporates analysis of alternatives; (5) Demonstrates customer relevance and addresses implications; (6) Uses clear and logical argumentation; (7) Explains change to or consistency of analytic judgments; (8) Makes accurate judgments and assessments; and (9) Incorporates effective visual information where appropriate. To ensure compliance with these standards, an office for Analytic Integrity and Standards (AIS) was established in the Office of the Director of National Intelligence.

Nevertheless, the belief that compliance with these standards would improve analysis and reduce uncertainty has been challenged from two directions. First, it has been claimed the tradecraft standards in ICD203 add nothing new to the existing practice (Lowenthal, 2012; Marchio, 2014; Gentry, 2015), and second, that reports complying with these standards may not produce more accurate estimates (Tetlock and Mellers, 2011). In this paper we investigate a third, more fundamental, issue that has so far received very little attention: whether the tradecraft standards can be reliably applied; that is, will two (or more) assessors evaluating the same report reach the same conclusions regarding its quality? (see Marcoci et al., 2018).

There is reason to be concerned. First, research into the design and implementation of assessment standards and requirements in higher education show consistently that standards expressed in linguistic terms are “fuzzy” and subject to multiple interpretations even by experienced evaluators (Sadler, 1987; Freeman and Lewis, 1998; Webster et al., 2000; O’donovan et al., 2004). ICD203 shares many of the characteristics of assessment standards in higher education.

Second, individual expert judgments in many related fields are routinely insufficiently reliable for practical applications. The reliability of judgments about facts and future events correlates poorly or not at all with the personal attributes that conventionally are associated with an expert’s credibility such as qualifications, years of experience, memberships, publications, or the esteem in which they are held by their peers. Cooke (1991) was one of the first to explore the ramifications of expert uncertainty for safety systems in engineering. Since that seminal work, hundreds of publications in spheres ranging from medicine and ecology to safety engineering and geoscience have documented the difficulties of identifying the attributes of reliable raters and the benefits of using group judgments to improve reliability (Burgman, 2015).

We could not find any evidence of attempts to identify the best assessors of analytic products or any research into the impact of training on their performance. To our knowledge, the reliability of the analytic tradecraft standards has not been systematically assessed. Yet, the question of reliability logically precedes investigation about its construct validity (do the standards really capture good quality of reasoning?), and predictive validity (does a good report make accurate predictions about the state of the world?). If the standards are not construed and used in the same way by different users, then the question of whether they engender more accurate estimates becomes moot. In this paper we report on the results of an experiment gauging the reliability with which the tradecraft standards in ICD203 can be applied. As noted above, ICD203 is meant to direct both the production and evaluation of analytic reports for quality control. For purposes of this experiment we focus on the latter aspect of ICD203.

## Materials and Methods

### Participants

We recruited 15 participants through an advertisement posted on the University of Melbourne’s School of Historical and Philosophical Studies mailing list. Selection criteria included: (1) completed or currently enrolled in a research higher degree in Arts/Humanities, (2) experience marking essays, (3) interest in the study and availability/willingness to work under imposed time constraints. Participants were selected from a pool of applicants based on best fit to the selection criteria, and were remunerated for their time. Seven were male, seven female and one preferred not to specify. Their average age was 35.93 (SD = 9.14) years. Seven had completed either a Masters or a PhD in the Humanities, while the rest were current PhD candidates.

### Materials and Procedures

This study draws on materials developed in the *Crowdsourcing Evidence, Argumentation, Thinking and Evaluation (CREATE)* program, an active research program (2017–2020) run by the Intelligence Advanced Research Projects Activity (IARPA).

#### Reports

Participants (henceforth: “raters”) were asked to evaluate the quality of reasoning of a set of hypothetical intelligence reports. The reports were generated by another group of research participants involved in testing a new online collaborative reasoning platform developed at the University of Melbourne, Australia, as part of the CREATE program. This platform (described in van Gelder and de Rozario, 2017), aims to (a) use the power of distributed processing within a network of individual thinkers, and (b) improve reasoning quality and the aggregation of solutions into a final, agreed solution. Users on the platform are requested to write individual analytical reports that outline the outcome (the solution to the problem) and the process (the underlying reasoning). They are invited to comment on one another’s contributions and update their own contribution in response to comments. The platform also encourages users to rate others’ solutions and the average quality rating (on a scale 0–100) determines the rank of each solution. The top-rated solution becomes the template for a final draft report, which is edited and ultimately submitted as a team report.

We collected 64 such reports generated by both individual users and teams in response to four different reasoning problems. All problems emulated reasoning challenges in real intelligence problems, with the exception that the problems were self-contained, i.e., all necessary information for solving them was contained in the problem description and the contextual information provided. Reports were generated during “beta-testing” of the platform in late 2017; test users included platform developer team members, junior analysts from an intelligence organization and individuals recruited online via targeted Facebook advertising. For the purposes of the current study, reports were downloaded from the platform and formats retained apart from minor changes such as removing specific references to and comments on other users’ submissions, so that each report could be analyzed as a stand-alone item. An example problem and report are provided in Supplementary Material.

The number of reports included in this study was determined prior to data collection. Given our knowledge of the internal procedure in AIS, we decided to measure the inter-rater (rather than intra-rater) reliability of the tradecraft standards using intra-class correlations (ICC). As the precision of ICC estimates depends on the number of raters, the number of reports and the true ICC, the numbers of included raters and reports were determined *a priori* to ensure sufficient precision (i.e., narrow confidence intervals) in our estimates of the ICC following Bonett (2002). With 64 products and 15 raters we were certain to have a 95% CI width (distance between the upper and lower bound) of no larger than 0.19 for the average ICC, and only slightly wider intervals in our estimates of the ICC for fewer raters (e.g., at worst 0.25 for estimating the ICC with four raters), regardless of the true ICC (Bonett, 2002).

#### Quality of Reasoning Rubric

ICD203 outlines the standards for good reasoning in intelligence analysis. These standards are operationalized by AIS in a “Rating Scale for Evaluating Analytic Tradecraft Standards,” an assessment rubric with nine criteria (Table 1). The rubric is very detailed. Every criterion has a short explanation regarding its scope. For example, “Criterion 4 – Incorporates analysis of alternatives” gives a paragraph of explanation detailing what it takes for a report to “incorporate analysis of alternatives.” Further, every criterion includes a comprehensive description of four levels of performance quality, i.e., “poor,” “fair,” “good,” and “excellent,” except for criterion 7, which describes the quality of judgments present in the report as either “unclear,” “conditioned,” or “unconditioned.” Each level of performance contains detailed sub-criteria that a report must meet to count as having satisfied the criterion “up to that level.” Additionally, each criterion contains a “Notes” section that gives detailed examples, tips, hints and elements to “watch out for” when applying the rubric. These three elements (high level explanation, sub-criteria for each level of satisfaction, and the notes) sum to a detailed rubric.

**Table 1:** Criteria used for assessing quality of reasoning in the rating scale for evaluating analytic tradecraft standards.

Criterion	Description
1	Properly describes quality and credibility of underlying sources, data, and methodologies
2	Properly expresses and explains uncertainties associated with major analytic judgments
3	Properly distinguishes between underlying intelligence information and authors’ assumptions and judgments
4	Incorporates analysis of alternatives
5	Demonstrates relevance and addresses implications
6	Uses clear and logical argumentation
7	Makes accurate judgments and assessments
8	Incorporates effective visual information where appropriate

For the purposes of this study, we omitted the criterion “Explains change to or consistency of analytical judgments” because it requires the report writer to have an understanding of previous analyses, which is irrelevant for the kind of constrained reasoning problems we used in this study. Furthermore, we made a small number of minor textual changes to accommodate the participants’ lack of familiarity with the jargon used in the original document. Numeric values were assigned to each performance level (i.e., 0 for “poor,” 1 for “fair,” 2 for “good,” and 3 for “excellent,” except for criterion 7, where 0 was awarded for “unclear,” 1 for “conditioned” and 2 for “unconditioned”). Scores were summed to give a total mark out of 31 for each report.

### Procedures

All raters completed the rating of the 64 reports over the course of 4 working days, in supervised sessions held at Melbourne University. This allowed us to mitigate the risk of non-independent evaluation, manage rater fatigue, ensure that raters understood the instructions, and that each report was marked in full. Compliance with instructions was monitored by one of the authors (AK).

The rating process started with a 2-h training/calibration exercise led by one of the authors (AK). First, raters were given the Rating Scale to peruse, make notes and ask questions regarding anything that was unclear or ambiguous. Second, raters were given a copy of a sample hypothetical intelligence problem (not included in the experiment, but also drawn from the CREATE program) and again encouraged to peruse it and ask questions. Next, raters were split into five groups of three and asked to evaluate a sample report individually at first and then deliberate in their group to reach a consensus on its evaluation using the Rating Scale. Afterward, groups shared their evaluations, followed by a robust discussion that highlighted differences in the way each group had interpreted the rubric criteria. Through facilitated discussion, differences were resolved and raters reached a consensus on an interpretation of how the criteria should be applied. They repeated this process for another two sample reports on the same hypothetical intelligence problem. Finally, the group was given one last sample report to mark as individuals and the facilitator assessed whether the group was sufficiently calibrated. At the end of this process, raters appeared to have reached a shared praxis or understanding of how to apply the rubric to the types of reports they would be evaluating. Each rater was then presented with a bound book containing the 64 reports in randomized order to eliminate order effects. Raters indicated their assessments on a personal score sheet, and were instructed not to discuss the reports, the rubric or the ratings with each other (data collected is summarized in the Supplementary Material).

Upon completion of the 64 report ratings, we obtained feedback from the raters on their experience with the rubric, and how they thought it performed as an assessment tool of quality of reasoning. All participants completed a questionnaire consisting of 10 open-ended and 10 multiple-choice questions, and took part in a focus group session (3 h). Both the survey and the focus group explored what the raters thought did and did not work well, which criteria were difficult to apply and why, whether there were elements of good or bad reasoning that were not captured by the rubric, the user-friendliness of the rubric, and their confidence in applying the rubric for the assessment of reasoning quality (see Supplementary Material, Table 3 for the full list of questions).

### Analysis

#### Rubric Reliability

The AIS quality control procedure involves multiple assessors concurrently evaluating products on the basis of ICD203. This motivated the use of the inter-rater reliability as our primary metric for rating consistency. Inter-rater reliability of the summed total scores for each report was assessed via ICC, a commonly used metric for the reproducibility or “consistency” of quantitative measurements made by different observers rating the same object(s). ICC values lie between 0.0 and 1.0, with higher values corresponding to greater agreement between raters.

First, we used the IRR package in R to calculate ICC values using a Two-Way Random-Effects Model, which assumes that each object is rated by a different set of raters who were randomly chosen from a larger population of possible raters. The ICC value we report here reflects absolute agreement rather than simple consistency between raters. We report both the “average” ICC value and the “single” ICC value, which differ in their interpretation. Their use depends on how the measurement protocol will be conducted in actual application. The “single” ICC is an index for the reliability of the ratings of single raters; the “average” ICC is an index for the reliability of different raters averaged together. The latter always results in higher ICC estimates. If in future use of the rubric, the average value across a number of raters is used as the assessment basis, the relevant reliability metric would be the “average” ICC. Conversely, if in future applications of the rubric, a single rater conducts the actual assessment, the “single” ICC type is the relevant reportable metric, even though the reliability study involves two or more raters. Regardless of the type of ICC, values <0.40 indicate poor inter-rater agreement, between 0.40 and 0.59 fair agreement, between 0.60 and 0.74 good agreement and >0.75 excellent agreement (Cicchetti, 1994).

Second, we examined the internal consistency of the eight criteria that make up the rubric with Cronbach’s Alpha. We also assessed item-total correlations to examine which (if any) criteria showed poor consistency with the rest of the rubric. Criteria with poor item-total correlations should be considered for removal from the rubric as they compromise reliability.

#### Rater Feedback

Results from the rater survey were summarized with descriptive statistics by one of the authors (BW). With regard to the focus group, two of the authors (AK, MM) independently coded the transcript and extracted the main themes. The resulting themes were reviewed by four authors (AK, AM, AV, and MM) to ensure that each theme was internally coherent, themes were distinct, and to reach consensus on their naming and interpretation.

## Results

### Inter-Rater Reliability

We calculated the ICC value for groups of raters of varying size. We first examined the “average ICC” metric for groups of between 2 and 15 raters. The “average ICC” provides a valuable estimate of reliability if future applications of the rubric involve aggregated evaluations, that is, if multiple raters are tasked with assessing single reports and their scores are averaged to produce a final quality assessment.

**FIGURE 1:**
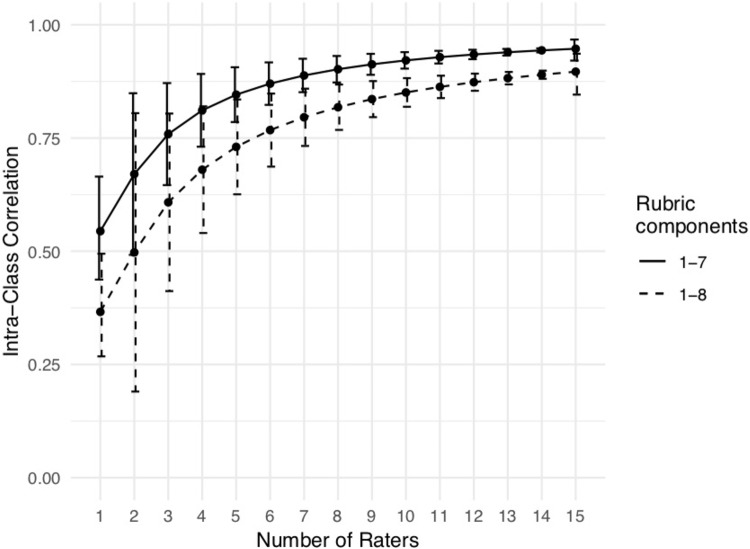
Relationship between number of raters and the Intra-Class Correlation (ICC), using either components 1–8 of the rubric, or only components 1–7. For *n* = 1 rater, the ICC estimate is based on an estimate of “single ICC” as calculated by the IRR package, using data from all 15 raters. For *n* = 2 to *n* = 15, to obtain the most precise estimates, we calculated the “average ICC” for all subsets of raters of size *n*, and took the mean. Error bars are 95% confidence intervals. For *n* = 1 and *n* = 15, the 95% confidence interval was calculated by the IRR package when estimating the ICC value. For *n* = 2 to *n* = 14, the confidence intervals are 95% bootstrap intervals over the subsets of raters.

To ensure reliability of our findings, we iterated over all possible subsets of each given group size *n*, and report the average ICC values for each *n*. We found an increase in reliability with increasing numbers of raters, starting from fair reliability with *n* = 2 raters [ICC = 0.498, 95% bootstrap CI = (0.196, 0.799)] to close to perfect reliability [ICC = 0.897, 95% CI = (0.846, 0.936)] when *n* = 15 raters were included (Figure 1). However, even a small set of three raters produces borderline good reliability [ICC = 0.608, 95% bootstrap CI = (0.416, 0.800)]. On the other hand, the “single ICC” metric produces an estimate of the reliability of a scale if just one rater scored it on a single occasion. Accordingly, this value does not depend on number of raters in the group, and we find that based on the 15 available raters, the single ICC value was poor [ICC = 0.366, 95% CI = (0.268, 0.495)].

Removing criterion 8 (“Incorporates effective visual information where appropriate”) from the overall score calculation improved the ICC values almost as much as doubling the number of raters (For example, using the complete rubric, three raters have an ICC of 0.608. Using six raters would improve the ICC to 0.768, whereas keeping only three raters but dropping criterion 8 improves the ICC to 0.759).

**FIGURE 2:**
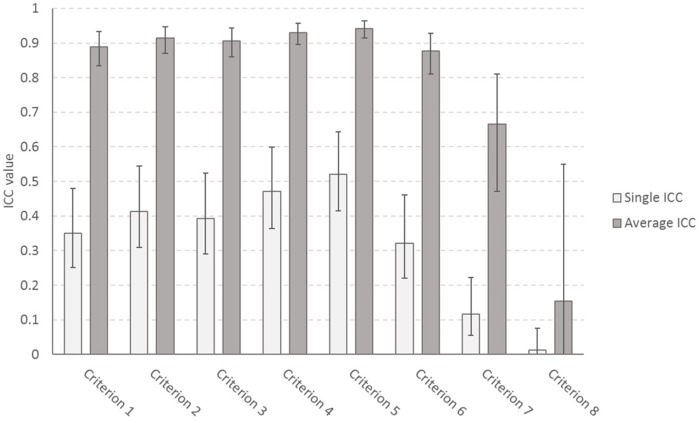
Intra-class correlation coefficients for each of the criteria of the assessment rubric based, with 95% confidence intervals.

**FIGURE 3:**
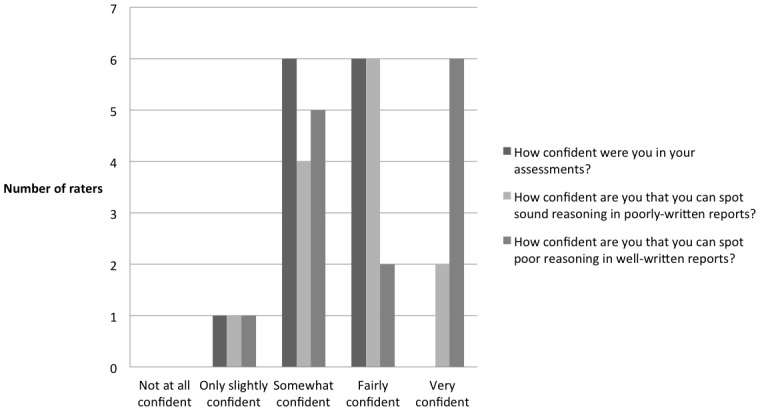
Raters’ evaluation of overall confidence in their report assessments (*n* = 13).

We also assessed the inter-rater reliability for individual criteria. The average ICC for 15 raters was excellent for all but criterion 8. The single ICC value for 15 raters, however, was poor to fair across the criteria (Figure 2). For a detailed analysis of the ICC values for all possible group sizes, see Supplementary Material (Figure 3).

### Internal Consistency

We calculated Cronbach’s Alpha separately for each of the 64 reports. The average value of Alpha across all reports was 0.606, which is considered “questionable” internal consistency. To examine which of the criteria might be responsible for the low consistency, we conducted an item-total correlation analysis (Table 2). This revealed that criteria 7 (“Makes accurate judgments and assessments”) and use 8 showed little correlation with the remaining items, and that removing them would improve the internal consistency of the rubric, with Alpha = 0.64 and 0.66, respectively. Rerunning the internal consistency analysis with both items seven and eight removed, revealed that this increased the internal consistency across all reports to “acceptable,” Alpha = 0.71.

**Table 2:** Item-total correlations for the eight criteria, and estimated Alpha values if the criterion were removed from the rubric.

Criterion	Item-total correlation	Cronbach’s Alpha, if deleted
Criterion 1	0.41	0.58
Criterion 2	0.46	0.57
Criterion 3	0.51	0.56
Criterion 4	0.38	0.59
Criterion 5	0.40	0.57
Criterion 6	0.53	0.55
Criterion 7	0.19	0.64
Criterion 8	0.09	0.66

The removal of criterion 8 in particular appears to be defensible as the inter-rater reliability for this criterion was very low, and it was identified in our qualitative analysis as neither critical to “good reasoning” nor transparent in its application, at least with reference to the specific set of test reasoning problems in this study (refer to Supplementary Material, Figure 4 for further details).

### Qualitative Feedback

The results from the qualitative feedback reveal that some criteria are ambiguous and provide insufficient guidance, allowing for potential discrepancies in interpretation. Moreover, some criteria lack specificity, that is, raters perceived areas of overlap such that judgments on some criteria depended on and were affected by others. Some criteria, in particular 1 (“Properly describes quality and credibility of underlying sources, data, and methodologies”) and 2 (“Properly expresses and explains uncertainties associated with major analytic judgments”), were also considered to describe multiple distinct attributes, potentially leading to conflation of reasoning faults in the overall assessment.

The following themes emerged as the key points of concern regarding the rubric used in this experiment. Illustrative verbatim quotes from the transcript are included for each theme:

1. “Box-ticking.” In applying the rubric, marks are assigned for the presence of certain attributes rather than on the actual quality or effectiveness of these elements.

“But if Y provides what we would think would be very weak evidence for the truth of X then for a certain kind of box ticker that arguably would be enough to qualify it as good but for someone who’s perhaps more quality minded and perhaps is arguably inclined to go beyond the rubric they might say - no that counts as poor.”

“And I felt it rewarded just putting headings and separating information. It gave too much value to just distinguishing when it was done very blatantly and not very well.”

2. “Granularity.” Descriptions for the different levels of satisfaction for a given criterion were not precise enough to enable clear categorization between poor-fair-good-excellent.

“I often had trouble distinguishing between fair and good … I wanted a third option in between cause they might provide say 1 sentence that’s obviously little detail but if they go into 3 or 4 sentences I wouldn’t call it considerable but you have to go one or the other.”

3. “Specificity.” Some criteria were too dense and measured multiple attributes at once (that may diverge in a single report). This may also have led to perceived overlap between criteria.

“… I found that reports that did perform in the excellent category in criterion 3 usually automatically perform well in criterion 4 as well because the two things kind of go together.”

4. “Logical consistency.” A number of comments identified logical inconsistencies, or a lack of overall coherence in the criteria in how they addressed the overall goal of “good reasoning.”

*“*…*still scored really highly even though … wasn’t addressing the question at all, because it presented a clear analytic message*,…*did it really well, but it should be a problem if it’s not answering the actual question.”*

“… it’s a little bit inconsistent … in the notes it says you’re not to rate reports on writing style or editorial practices. Seems a bit contradictory. But also I do lot of work as an editor and I would argue that writing style and editorial practices are connected to clarity and logic of argument. The way you use words, punctuation could change the meaning of a sentence.”

5. “Aggregation.” The rubric provides no detail on how criterion scores are aggregated into a total score. Perceived weighting of the different criteria influenced rater behavior that may introduce inconsistency in the application of the rubric.

“If you’re going to use rubrics you need to understand how each criterion is weighted because some are literally more important than others …. It kind of depends on what the actual goal is as to how things are weighted.”

6. “Unfair penalization.” Raters felt forced to penalize a report for “going the extra mile,” rather than rewarding some risk taking.

*“It’s confusing that if you say you make a claim about probability and don’t explain it all you get fair whereas if you explain it wrongly*…*you get penalized.”*

Overall, raters reported being moderately confident in their report assessments, with the majority being “fairly” or “somewhat” confident (Figure 3).

When asked how useful (or not) the rubric is in helping to separate quality of reasoning with quality of writing (*n* = 14), eight found it to be “fairly useful” (57%), four found it to be “somewhat useful” (29%), and two found it “only slightly useful” (14%).

## Discussion

Our study clearly illustrates that the tradecraft standards and their operationalization in the AIS rubric present “poor” inter-rater reliability when deployed by individual raters and “good” to “excellent” (when criterion 8 is excluded) inter-rater reliability when deployed by groups of at least three raters. We expect this will be true of reports of the kind used in this study: i.e., those that are relatively simple and self-contained. In contrast, a group of 15 raters approaches perfect reliability. Therefore, our results suggest that evaluations completed by a single assessor on the basis of the tradecraft standards should be interpreted with extreme caution. This study focused exclusively on the role of the tradecraft standards in the *evaluation* of analytic reports. But the low inter-rater reliability of the tradecraft standards when used by single raters also raises in our minds concerns regarding their use in the *production* of analytic reports.

Our findings further indicate that the criteria are not sufficiently precise, are ambiguous, may not be exhaustive in capturing the core elements of good reasoning, and may be perceived by analysts as not applicable in the development of a well-reasoned report. Users may also perceive the standards to be emphasizing “process” over “deep quality.” The criteria should therefore be revised to ensure that they are internally consistent and that each addresses a single issue. Moreover, in the absence of knowledge on how the criteria scores will be aggregated, raters may hold private and different weightings that influence their assessments of the products. Similar concerns may arise in the minds of the analysts who *produce* reports using ICD203. This may lead to a focus on report attributes that are of limited relevance to the overall quality or the accuracy of the analysis.

Further research is required to provide a comprehensive evaluation of the success of IRTPA and ODNI/AIS in creating a reliable and valid quality control process for the IC. This study is a first step in that direction and has its limitations.

First, note that a group’s rating of a report consists in the mathematical average of the individual ratings. This approach cancels out disagreements. Another approach would be to require discussion and/or third party moderating to resolve disagreements on the application of the standards before individual ratings are averaged. Whether such an approach would raise the reliability of teams of evaluators (and by how much) remains an open empirical question that we aim to address in future research. And the potential for bias should not be overlooked. Nevertheless, the fact that small (*n* = 3–4) teams can apply the standards consistently (even when using simple mathematical averaging) means teams of evaluators using the current AIS operationalization of ICD203 can perform reliable quality control.

Second, our raters were novices in the sense that they had no prior experience in using either the tradecraft standards or the AIS rubric. However, they had considerable experience using assessment rubrics in higher education and assessing written work, they were given training on the AIS rubric and underwent a calibration exercise. Given the strong parallels between rubrics in education and this one, there are no obvious reasons to expect that novice professional raters would perform appreciably better. Moreover, the literature on expertise teaches us that the attributes of reliable evaluators are very elusive (Burgman, 2015). So whether the results of the present study would hold for senior assessors on real intelligence reports remains an open (empirical) question. On the one hand, due to experience, they may be more consistent in the application of the standards. On the other, “real-world” (unconstrained) intelligence problems and reports would be more difficult to assess, and the impact of idiosyncratic understanding of the standards of analysis and biases should not be underestimated. It is unclear to us how to weigh these considerations *a priori* and we hope to address the reliability of the standards with senior assessors and on unconstrained intelligence problems in future research.

Third, we should also note that, all other factors being equal, ICC values are depressed when there is little variation in the objects being rated. If reports used in this study were relatively similar in quality, this would therefore impose an upper limit to the achievable ICC. However, the reports represented a range of products generated on the platform, by both individuals and teams, and vary in sophistication and reasoning quality. This was confirmed by examining the peer assessments produced by contributors themselves using the optional rating functionality on the platform. While only a subset of reports (*N* = 40) received peer-ratings, the quality assessments ranged from 10 to 85 on a 100-point scale (*M* = 57.4, *SD* = 14.45), suggesting sufficient variation in quality for the purposes of ICC calculation. Future evaluation of the tradecraft standards should nevertheless be performed on a wide range of reports varying in style, purpose and quality.

Furthermore, whether *accurate* quality control is possible on these standards remains an important open question. Just because averages of groups of three or more raters are consistent does not mean that their assessments accurately capture the true quality of reasoning. This is a matter of external validity. Validity is dependent on reliability – an unreliable instrument cannot make accurate measurements – hence, this study should be considered a first step toward an investigation of the validity of the tradecraft standards. But the matter of whether these standards and the associated rubric used by AIS is actually a valid indicator of quality of reasoning, and whether a report that rates highly is also producing the “correct” results is one for future study.

Finally, in-depth analysis of the 15 raters’ experience in applying the rubric revealed potential leverage points to revise the instrument with a view to increasing its internal consistency. Some criteria were too prescriptive, described as a “box-ticking exercise,” leading to frustration. Raters felt that if they complied with the rubric, they were forced to unfairly penalize genuine, though incomplete, analytical process, whereas the absence of analytic effort was rewarded, comparatively. In a context where analysts may already feel under pressure to align with a preferred narrative (Stimson and Habeck, 2016), this may promote a culture of conservative analytical approaches at the expense of appropriate risk-taking, which may be detrimental to the overall quality of reports.

Commenting on the intelligence reform brought about by IRTPA, Robert Cardillo (2010), who served as the Deputy Director of National Intelligence for Intelligence Integration, wrote that ICD203 “injects rigor into our processes and products and holds analysts and managers accountable for results” (2010: 44), i.e., by providing a tool for assessing the analytic products they generate. The results of the present study suggest that this optimism may be compromised when evaluations are undertaken by single assessors, but that it may be vindicated by teams who can consistently apply the tradecraft standards to evaluate the quality of products generated by the IC.

## Ethics Statement

This research project has been approved by the Human Research Ethics Committee of The University of Melbourne, with ethics ID number 1646872.5. All participants in this study were employed as casual research assistants and signed a work contract. Participants who produced the reports used in this study have signed an informed consent form.

## Author Contributions

AM and AV designed the experiments with the contributions of MB, FST, HF, BW, and FF. AK conducted the experiments. AK and MM coded the transcripts from the focus group, and BW analyzed the survey data. AV and ES analyzed the quantitative data. AM and AV took the lead in writing the manuscript. All authors provided critical feedback and helped to shape the research, analysis and manuscript.

## Conflict of Interest Statement

The authors declare that the research was conducted in the absence of any commercial or financial relationships that could be construed as a potential conflict of interest.
