# Supplementary material for: Better Together: Reliable Application of the Post-9/11 and Post-Iraq US Intelligence Tradecraft Standards Requires Collective Analysis
Source: Front Psychol. 2019 Jan 7;9:2634. doi: 10.3389/fpsyg.2018.02634 (PMC6330287; doi:10.3389/fpsyg.2018.02634)
Supplement: Supplementary file 1 [file Data_Sheet_1.PDF]

Kalukistan Bomb Problem

SPECIAL OPERATIONS COMMAND ASIA

MEMORANDOM TO THE ID2 OFFICER, SOCA – KALUKISTAN.

SUBJECT: Recent Car-bombing of Ministry of Minerals and Mines. Suspects in National Gendarmerie detention.

NOTE TO PROBLEM-SOLVER(S)- you are not required to have a prior understanding of forensics or DNA science and the necessary information to solve this problem can be found in these pages.

As an Identity Intelligence (ID2) Officer serving with Special Operations Command Asia (SOCA) and currently stationed in Kalukistan, your role is liaising with and providing training and assistance to the Central Government's National Security Forces and the local police (gendarmerie) in the Capital.

Kalukistan is a Central Asian country with vast mineral wealth, a weak central government and a history of corruption and endemic inter-tribal/ethnic warfare. Historically and geographically at the crossroads of trade routes and imperial ambitions, it also has a long history of military incursions and occupations by numerous foreign powers over the last century.

Most recently, the Central Government has aligned itself with a coalition of Western Nations who have been providing military and economic assistance as part of the global 'War on Terror'. In the capital city of Khyberadad the population is on edge as the spring, traditionally the beginning of the fighting season, approaches. The Kalukistan National Security Forces are stretched thin, but have increasingly been supported in the field by Coalition air power and on the ground by Special Operations troops.

In Khyberadad, the National Gendarmerie (NG) plays the role of police and security force. Over the last year, the NG has been implementing a biometric identity program and DNA database in cooperation with and assisted by the Coalition advisors, and in particular the Special Operations Command Asia's Identity Intelligence taskforce of which you are a part.

Initially implemented for all government employees and employees of Western Aid Agencies and Economic Development Projects (particularly in the resource extraction industries), the National Biometric program has the goal of creating a file for all of the Khyberadad's residents, though the database is far from complete at the present time.

Several days ago, at 7:00 p.m., a large car bomb exploded outside the Ministry of Minerals and Mines on the outskirts of the central Embassy District. At the time, a large delegation from Western multi-nationals was meeting at the Ministry to discuss access to mineral deposits in the remote Eastern provinces. The bomb failed to collapse the building, and none of the delegates was killed, but there were multiple injuries and casualties among the building's workers.

Signals intelligence has confirmed that the bomb was detonated remotely using a cell phone, but as the phone used was a ‘burner’ there has been little progress on this front. Unfortunately, the building CCTV system had been out of service for several days and there is no footage of the car arriving on the scene. However, a drone doing a sweep of the city has footage showing the car parked on the scene around 6:30 p.m.

Over the last two years, in response to other attacks, the National Gendarmerie has performed a number of sweeps in the capital, arresting large numbers of people, detaining and interrogating them. Most of those interrogated were quickly released, but some were transferred to a special facility operated by SOCA, and there is widespread discontent among the population concerning this, as well as the sweeps more generally. The government and the security forces still have tepid support from the population, despite widespread resentment of corruption and Western interference.

Two days ago, acting on a human intelligence source, the National Gendarmerie raided Oasis Telecom, an internet café and telecoms shop in the crowded and impoverished Khana Girad district. Like many such telecom shops, Oasis Telecom provided a range of services to its customers. The shop bought, sold and repaired mobile phones, sold SIM cards and telephone cards for international calls, and offered a variety of money transfer services. The money transfer services were popular with the large number of low-income foreign workers living in the district, who relied on these services to send money back to their families in their countries of origin.

National Gendarmerie operatives found a bomb-making workshop in the basement of Oasis Telecom, which was accessible through the owner’s office, and through a separate back entrance. Based on the evidence found in the workshop, several arrests have been made.

ACTIONS REQUIRED BY ID2 OFFICER

Your task is to assess the evidence from the National Gendarmerie, and recommend *one* of the actions listed below be carried out for each of the four suspects apprehended (Suspects A-D, codenamed “The Owner”, “The Courier”, “The Old Friend”, and “The Customer” respectively). Justify your recommended action. All the information you need to make and justify your decision is contained in the following documents.

Action 1: Complete a full biometric identity file and recommend release. Leave decisions concerning detention/release and any logistics regarding further surveillance to the National Gendarmerie.

Action 2: Recommend the release of the suspect after completing a full biometric identity file. Send a request for active signals surveillance of the suspect to the SOCA/ Intelligence Directorate Command, but do not request human intelligence assets for active surveillance of the suspect.

Action 3: After completing a full biometric identity file, release the suspect and request active human surveillance of the suspect as well as active signals surveillance.

Action 4: After completing a full biometric identity file, request transfer of the suspect to the Secure SOCA facility for interrogation and processing.

SPECIAL OPERATIONS COMMAND ASIA (SOCA)

MEMORANDUM FOR ID2 OFFICERS, DIRECTORATE OF INTELLIGENCE, SENIOR COMMAND

SUBJECT: Changes to interrogation and surveillance protocols in Kalukistan

In the interests of reducing recent unrest in and around Khyberadad and improving the status of the National Gendarmerie (NG) with the local population, SOCA has made the following changes to interrogation and surveillance protocols:

- Terror suspects should only be transferred to the SOCA secure site outside of Khyberadad for comprehensive questioning by SOCA operatives when it is highly likely the suspect is involved in terrorist activity. All other suspects should be left in the custody of the NG for decisions regarding further detention or release.
- A realignment of budget priorities has restricted SOCA's capacity to provide active signals intelligence assets for suspect-specific monitoring and (especially) human surveillance assets for monitoring/observing terror suspects. This means that previous strategies of blanket surveillance are no longer able to be accommodated, and so local ID2 personnel are expected to allocate surveillance assets as efficiently as possible. Any request for SOCA resources for ongoing surveillance is expected to be able to be fully justified to SOCA Senior Command.

It is vital these changes to protocol be adhered to. Increases in the local population unrest, and the continued erosion of trust in local law enforcement institutions due to the appearance of being beholden to foreign interests are jeopardising the broader SOCA mission in Kalukistan beyond the identification and capture of terrorists.

National Gendarmerie, Khyberadad Office

File 13701

Suspect A: "The Owner"

Background:

45 year old male, average height, overweight. Dark hair, grey-green eyes. Wears vision-correcting glasses. Unmarried. Lives alone in west Khyberadad. Owner and manager of Oasis Telecom. Previously worked in civil engineering and has verified experience with explosives.

Forensics lab notes:

DNA material obtained from the computer keyboard, the mobile phone and from the soldering iron in bomb factory are a match for the Owner—see forensics lab report.

Interview notes:

- Claims he was at working at the Oasis Telecom/Internet Café at the time of the bombing, along with Suspect B (the Courier) until 9:00 pm. Asked if he had left the shop, he replied that he had: once to buy lunch and another time 'in the evening, to get some exercise and a coffee'.
    - UPDATE: Local café worker confirms that the Owner and friends frequently come in, but couldn't specify if the Owner had been there on the day of the bombing.
- Also claims that 6 months previously that he had rented the basement where the bomb factory was located, to two foreign workers who came and went through the back alley door and thus unseen and generally unheard by him. He only knew their first names, which are such common names as to be useless, describing them only as average height, dark hair and complexion. He claims he had rented the basement out many times in the past and didn't ask his renters questions as long as they paid cash for two months up front and paid rent on time.
    - UPDATE: There is no sign of these two alleged foreign workers. However, DNA evidence suggests that multiple unknown people may have used the basement and been involved in the bomb manufacturing.
- When asked about Suspect C (the Old Friend), he claims they are old friends from the same village in the south of Kalukistan and admits he had dinner with the Old Friend the night before the bombing at a restaurant near Oasis Telecom where they shared some food, drank some coffee and shared a hookah pipe. The Owner claims that after dinner, he left to go close up Oasis Telecom and then went home, but did not then, or at any time in the last 6 months, enter the basement.

National Gendarmerie, Khyberadad office

File 13702

SUSPECT B: "The Courier"

Background:

22 year old male. Has worked at Oasis Telecom for approximately three years as a store attendant and courier. Owns a motorcycle, used for courier jobs. Unmarried. Lives with parents and siblings in West Khyberadad.

Forensics lab notes:

The Courier's DNA matched to DNA collected from the mobile phone and computer keyboard in bomb workshop—see forensics report.

Interview notes:

- Asked about whereabouts on the evening of the bombing. The Courier claims to have been at Oasis Telecom all afternoon and evening until close at 9:00pm, and then rode motorbike home, where he stayed all night with his family.
    - UPDATE: The Courier's family interviewed at their home. The Courier's father claims that the Courier arrived home after 9:00pm on the day of the bombing.
- Asked about relationship to the Owner. The Courier claims he is just an employee of the Owner; they are not close and do not associate outside of work. The Courier only tends the shop and makes deliveries around the city on behalf of the Owner.
- Asked about knowledge of the whereabouts of the Owner on the evening of bombing. The Courier says the Owner left the store on the day of the bombing, but can't remember the precise times he left and returned. The Courier claims that the Owner will often leave the store for extended periods,

especially in the evenings, to spend time eating and drinking with friends and associates. The Courier claims not to know who any of these friends and associates are.

- Asked about knowledge of the bomb-making workshop in the basement of Oasis Telecom. The Courier claims no knowledge of the workshop; he has never had any need to go that far out the back of the store building.
- Asked about the computer keyboard found in the bomb-making workshop. The Courier claims to know nothing about it, and maintains he has never been to the basement of the Oasis Telecom. The Courier volunteers that the computer on the store counter was replaced with a new one a few weeks ago by the Owner, and the old computer was removed. The Courier claims not to know what happened to the old computer.
    - UPDATE: Examination of the computer found in bomb-making workshop strongly indicates that this was the old computer formerly on the store counter – the computer contained files and point-of-sale software consistent with being used in retail sale.
- Asked about connection to Suspect C (The Old Friend). The Courier claims he has never met the Old Friend.
- Asked about connection to Suspect D (The Customer). The Courier said he recognised the Customer as an occasional customer, but had no relationship with the Customer other than that. He remembers the Customer coming in to sell his mobile phone.
- Asked about mobile phone. The Courier said he recognised it as the phone that the Customer had sold back to Oasis Telecom. The Courier said he did not know what had become of the mobile phone after that.
- The Courier maintains his innocence, claims he had no knowledge of a bomb-making facility at the Oasis Telecom, and claims he has nothing to do with the bombing.

<u>National Gendarmerie, Khyberadad office</u>

File 13703

<u>SUSPECT C: "The Old Friend"</u>

Background:

46 year old male. Warehouse manager at Khyberadad airport, employee of multinational corporation International Freight Solutions. Married. Lives with wife, 45, in South Khyberadad. One daughter, currently studying at university overseas. Does not own or drive a car. Arrested three years ago at an anti-Western demonstration, but claimed he was just a passer-by caught up by mistake.

Forensics lab notes:

The Old Friend's DNA profile was added to the national DNA register last year. The Old Friend's DNA matched to DNA collected from soldering iron in bomb workshop—see forensics lab report.

Interview notes:

- Asked about whereabouts on the evening of the bombing. The Old Friend claimed that he met up with his wife at the end of her shift at her workplace (grain processing factory) at 6:00pm, and they took the local bus home together, arriving home at approximately 7:00pm, and then remained there for the rest of the night.
    - UPDATE: wife's employment at grain processing factory confirmed with factory management.

    - UPDATE: A SOCA surveillance drone monitoring Khyberadad passed over the industrial area at 6:00pm. Footage indicates two people, likely a male and female, leaving the grain processing factory and walking in the direction of the bus stop on a nearby main road. Identities of two people could not be determined.
- Asked about connection to Suspect A (The Owner). The Old Friend claims the two are old friends, they grew up in the same village in the south of Kalukistan and attended the same polytechnic.
- Asked about the last time the Old Friend saw the Owner. The Old Friend admitted the two had seen each other the night before the bombing. They met at a restaurant close to Oasis Telecom, and shared a plate of bread and meat, then had coffee and shared a hookah pipe. The Old Friend claims that after dinner, the Owner said he was going to back to Oasis Telecom to close up. The Old Friend claims he went straight home on the bus, and did not go to the Oasis Telecom with the Owner.
- Asked about connection to Suspect B (The Courier). The Old Friend claims that he has never met the Courier.
- Asked about connection to Suspect D (The Customer). The Old Friend claims that he has never met the Customer.
- The Old Friend maintains his innocence, claims he had no knowledge of the bomb-making facility at Oasis Telecom, and claims he has nothing to do with the bombing.

National Gendarmerie, Khyberadad Office

File: 13704

Suspect D: "The Customer"

Background:

Male aged 30, average height. Dark hair, dark eyes. Dark beard. Married. Lives with wife and mother in law in east Khyberadad. Owns and runs a barbershop.

Forensic lab notes:

DNA material obtained from the mobile phone—see forensics lab report.

Interview Notes:

- The Customer claims he was at working at his barbershop at the time of the bombing.
- When questioned about the presence of his DNA on the phone, he claims he sold the phone to Oasis Telecom, buying a newer used phone at the same time. He claims the transaction took place with Suspect B (The Courier). He also states he was a frequent customer of the shop for its internet and telecom services.
    - UPDATE: Phone records/signals intelligence suggest that the Customer did indeed buy a new phone on the day he claims.
- The Customer maintains his innocence, claims he had no knowledge of the bomb-making facility at Oasis Telecom, and claims he has nothing to do with the bombing.

National Gendarmerie Forensic Biology Laboratory Report (Khyderabad)

EXAMINATION AND SEROLOGICAL ANALYSIS

Items submitted:

The following items were found in the basement of the Telecom Shop and submitted for a forensic biology analysis.

Item 1: mobile phone

The mobile phone was examined for the presence of human genetic material. The following samples was collected and assigned and exhibit number:

Exhibit number	Method
EX01	Vacuum-swabbing

Item 2: Computer Keyboard

The computer keyboard was examined for the presence of human genetic material. The following sample was collected and assigned and exhibit number:

Exhibit number	Method
EX02	Vacuum-swabbing

Item 3: Soldering Iron

The soldering iron was examined for the presence of human genetic material. The following sample was collected and assigned and exhibit number:

Exhibit number	Method
EX03	Vacuum-swabbing

DNA ANALYSIS

Samples were tested by deoxyribonucleic acid (DNA) analysis of short tandem repeats (STRs) on 13 autosomal STR loci using polymerase chain reaction (PCR) analysis. The raw analytical data is available upon request.

RESULTS

DNA extracted from exhibit EX01 provides matches for THE OWNER, THE COURIER and THE CUSTOMER.

DNA extracted from exhibit EX02 provides matches for THE OWNER and THE COURIER. Analysis also identified genetic material from at least 2 other individuals (not found in National Biometric Database).

DNA extracted from EX03 provides matches for THE OWNER and THE OLD FRIEND.

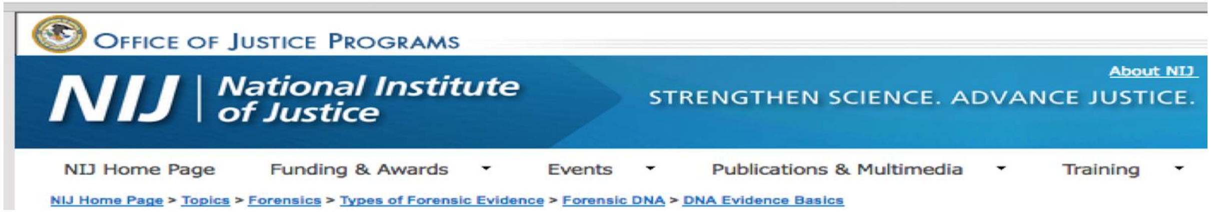

Overview of Steps in Analyzing DNA Evidence

Several basic steps are performed during DNA testing regardless of the type of test being done. The general procedure includes:

1) the isolation of the DNA from an evidence sample containing DNA of unknown origin, and generally at a later time, the isolation of DNA from a sample (e.g., blood) from a known individual;

2) the processing of the DNA so that test results may be obtained;

3) the determination of the DNA test results (or types), from specific regions of the DNA; and

4) the comparison and interpretation of the test results from the unknown and known samples to determine whether the known individual is not the source of the DNA or is included as a possible source of the DNA.

Any probative biological sample that has been stored dry or frozen, regardless of age, may be considered for DNA analysis.

Each additional test at a previously untested locus (location or site) in the DNA provides another opportunity for the result of "exclusion" if the known individual being used for comparison is not the source of the DNA from an evidence sample of unknown origin. If, however, the known individual is the source of the DNA on the evidence sample, additional testing will continue only to include that individual as a possible source of the DNA. When a sufficient number of tests has been performed in which an individual cannot be excluded as the source of the DNA by any of the tests, a point is reached at which the tests have excluded virtually the entire world's population and the unique identification of that individual as the source of the DNA has been achieved.

Steps in DNA Sample Processing

The following is a review of the steps involved in processing forensic DNA samples with STR markers. STRs are a smaller version of the VNTR sequences first described by Dr. Alec Jeffreys in the 1980s. Samples obtained from crime scenes or paternity investigations are subjected to defined processes involving biology, technology, and genetics.

Biology

Following collection of biological material from a crime scene or paternity investigation, the DNA is first extracted from its biological source material and then measured to evaluate the quantity of DNA recovered. After isolating the DNA from its cells, specific regions are copied with a technique known as the polymerase chain reaction, or PCR. PCR produces millions of copies for each DNA segment of interest and thus permits very minute amounts of DNA to be examined. Multiple STR regions can be examined simultaneously to increase the informativeness of the DNA test.

Technology

The resulting PCR products are then separated and detected in order to characterize the STR region being examined. The separation methods used today include slab gel and capillary electrophoresis (CE). Fluorescence detection methods have greatly aided the sensitivity and ease of measuring PCR-amplified STR alleles. After detecting the STR alleles, the number of repeats in a DNA sequence is determined, a process known as sample genotyping.

The specific methods used for DNA typing are validated by individual laboratories to ensure that reliable results are obtained and before new technologies are implemented. DNA databases are valuable tools and will continue to play an important role in law enforcement efforts.

(This text is adapted from https://nij.gov/topics/forensics/evidence/dna/basics/pages/analyzing.aspx and is in the public domain.)

Short Tandem Repeat (STR) Analysis for DNA Profiling

A common technique for identifying DNA profiles is short tandem repeat (STR) analysis. While human beings share more than 99% genetic similarity, STR analysis exploits the fact that there are specific regions (termed ‘loci’) of nuclear DNA, known as “variable number short tandem repeats” that do not code for proteins and that are highly variable between individuals. Autosomal chromosomes are those chromosomes which are not involved in determining a person's gender. STRs on these autosomal chromosomes are known as autosomal STRs and 13 of these autosomal STR’s serve as the basis for forensic analysis in most countries, including the United States[1]. If these 13 loci are examined, the chance that any two individuals (who are not identical twins) will have the exact same DNA profile at all loci is approximately one in a billion[2].Thus, by comparing DNA profiles at a number of loci, it can be determined to an extremely high level of confidence whether the DNA profiles match or not.

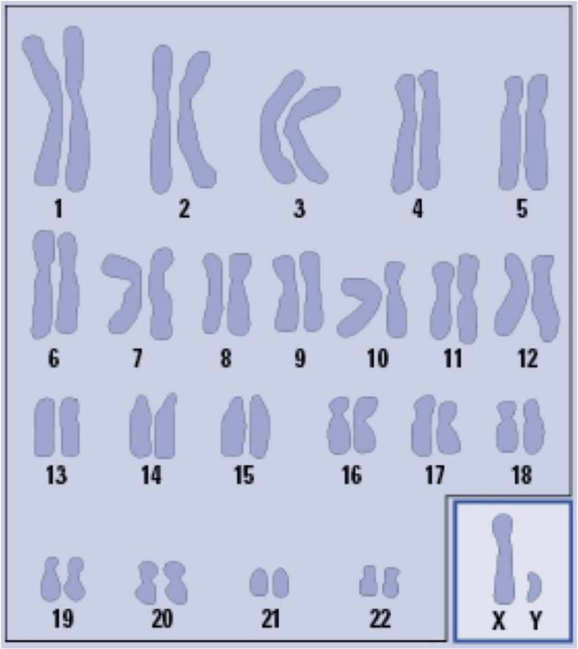

Figure 1 Human beings have 23 sets of chromosomes. 22 sets are known as autosomal chromosomes, and the 23 pair, the X and Y chromosomes, determine gender. Adapted from nij.gov.

It is advantageous to select a standard set of loci to be used by all forensic analysts, since this means that a common database of DNA profiles can be built up and shared. Any sample of DNA from an unknown individual can be analysed using STR and then compared to the known DNA profiles in the database to search for a match. SOCA has adopted a standard set of 13 loci to be used by all forensic analysts.

Polymerase Chain Reaction (PCR)

Polymerase chain reaction (PCR), sometimes referred to as 'molecular photocopying' uses the polymerase enzyme to replicate regions of DNA in a test tube- a process discovered by Dr. Kary B Mullis in 1983 for which he was later awarded a Nobel prize[3].

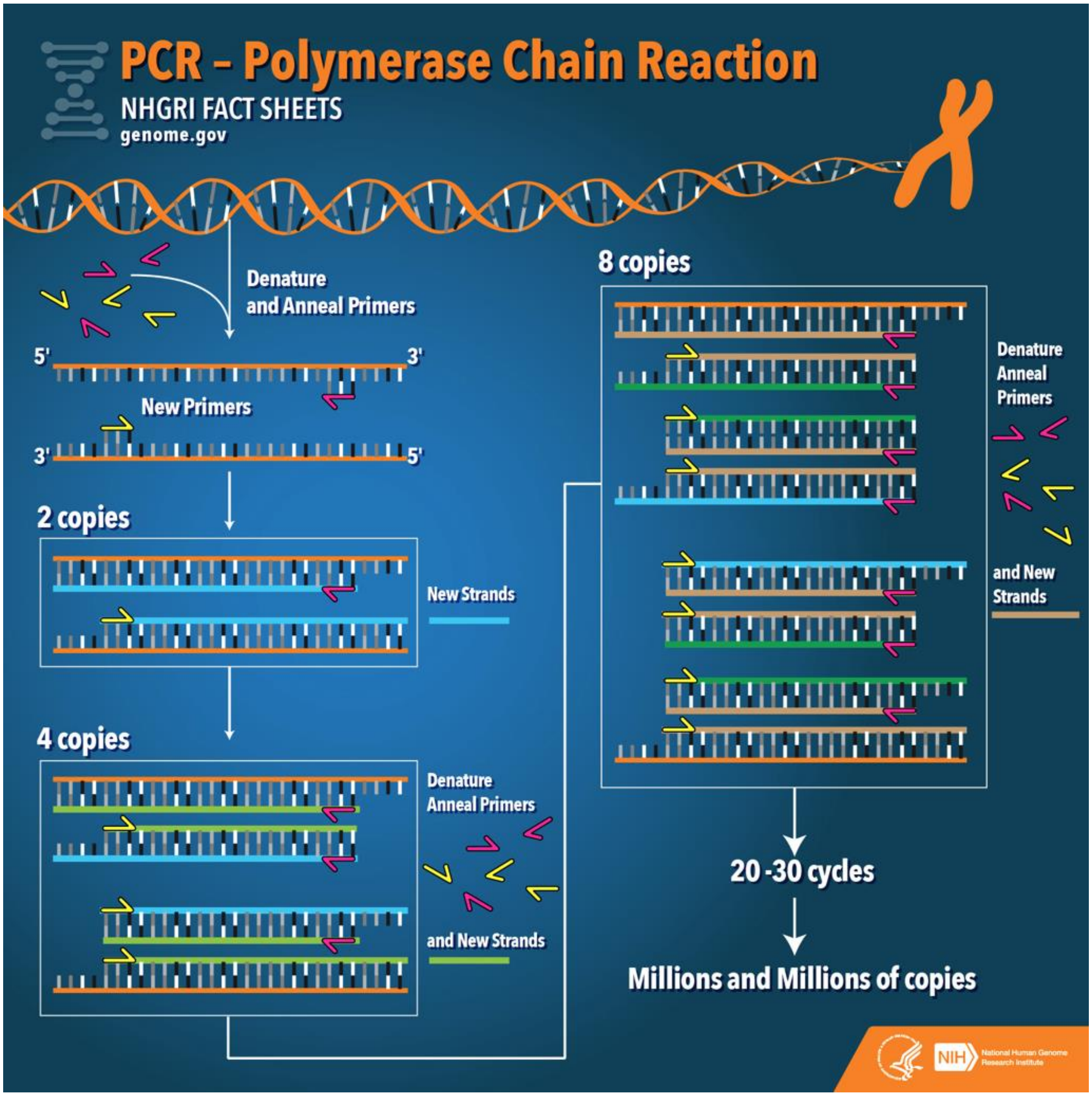

Figure 2 PCR Polymerase Chain Reaction, adapted from genome.gov

By repeating the copying process, small amounts of DNA can be amplified by orders of magnitude, resulting in billions of copies of the targeted DNA segments. In forensic investigations, minute quantities of DNA material can be obtained from a crime scene and compared to that from suspects or against a database. Careful identification, collection, and laboratory procedures are used to prevent contamination, which is an increased risk because of the small sample sizes and exponential amplification. Secondary DNA transfer, in which a person transfers DNA from another person, with whom they have had contact, to an object, has also been raised as an issue with the use of PCR. This can be problematic with regard to assumptions that the presence of small quantities of DNA at a scene is the result of a person's direct handling of an object and thus indicative of said person's presence at the scene[4]. Previously to the development of PCR, forensic analysis of DNA material required a large biological sample size – about the size of a bottle cap.

Because PCR can be used with samples as small as a few cells, it has even been successfully used to solve a number of cold cases using old and highly degraded evidence. In addition to genetic fingerprinting, DNA produced with PCR has many uses. Nearly all of the mapping of the Human Genome Project (HGP) was made possible using PCR[5]. Many other laboratory and clinical techniques use PCR. These include research on genetically modified organisms, the detection of microorganisms such as the Ebola virus, testing and diagnosis of inherited gene based disorders, and DNA fingerprinting.

Kary B Mullis and Michael Smith shared the 1993 Nobel Prize in Chemistry for Mullis' invention of PCR and Smith's related work on "... *fundamental contributions to the establishment of oligonucleotide-based, site-directed mutagenesis and its development for protein studies*[6]".

[1] Norrgard, K., "Forensics, DNA Fingerprinting, and CODIS" *Nature Education* 1(1) (2008). https://www.nature.com/scitable/topicpage/forensics-dna-fingerprinting-and-codis-736

[2] https://nij.gov/journals/267/pages/extending-str.aspx

[3] https://www.nobelprize.org/nobel_prizes/chemistry/laureates/1993/mullis-facts.html

[4]C. M. Cale *et al*. J. Forensic Sci. http://onlinelibrary.wiley.com/doi/10.1111/1556-4029.12894/abstract;jsessionid=4BEC44B11DEFA505B36A976C789AA214.f02t02

[5] genome.gov

[6] https://www.nobelprize.org/nobel_prizes/chemistry/laureates/1993/

EXAMPLE REPORT

The Owner of Oasis Telecom is implicated in bomb-making; we recommend he be detained and interrogated at the Secure SOCA Facility, with other suspects being placed under surveillance

Recommendations

We recommend Action 4 for suspect A, Action 3 for suspect C and Action 1 for suspects B and D.

There is a high likelihood that the Owner (Suspect A) of Oasis Telecom in Khana Girad district is involved in, or is complicit in, the manufacture of bombs in a workshop in his basement, and may have been responsible for the recent car bomb at the Ministry of Minerals and Mines. He should be transferred to SOCA for interrogation and processing (Action 4). His Old Friend (Suspect C), may be implicated and should undergo active human and signals surveillance (Action 3). A full biometric identity file should be completed for all four suspects (i.e. Action 1 for the Courier and the Customer). We recommend an ongoing investigation; there is currently no evidence connecting the bomb-making workshop to the car bombing.

Assignment

As Identity Intelligence (ID2) Officers serving with Special Operations Asia (SOCA) and currently stationed in Kalukistan our role is to liaise with, train and assist the Central Government's National Security Forces and local police, the National Gendarmerie, in the capital Khyberadad.

A car bombing recently occurred outside the Ministry of Minerals and Mines in Khyberadad, during a meeting on access to mineral deposits in Kalukistan. The meeting involved a a large delegation of Western multinationals. The explosion left delegates unharmed but left many casualties among the building's workers. Following the explosion, acting on a human intelligence source, the National Gendarmerie raided Oasis Telecom, an internet café and Telecom shop in the Khana Girad district. National Gendarmerie operatives found a bomb-making workshop in the basement of Oasis Telecom, which was accessible through the owner’s office and through a separate back entrance.

Based on the evidence found in the workshop the National Gendarmerie arrested four suspects.

We have been tasked with assessing the evidence of the National Gendarmerie and recommending one of four actions to be carried out for each of the four suspects. We were required to justify any recommendation for human or signal surveillance, due to SOCA's budget constraints and to use Action 4 (detention and interrogation) only when it is highly likely that the suspect is involved in terrorist activity.

Key Judgments

All probability assessments in this report express degrees of belief. Terms of estimative probability are defined according to the ICD 203 table.

1. The basement at Oasis Telecom has been used for making bombs (see Assumptions and Reasoning).
2. The Owner of Oasis Telecom (the Owner) rented the basement to the bomb-makers, and/or was directly involved.
3. The Owner is very likely (80-95%) to be a collaborator in the bomb-making workshop as he owns the premises and his shop was the source of some of the equipment which he did not deny.
4. It is likely (55-80%) that he was involved in the manufacture of a bomb as his DNA was on the soldering iron and he had the skills (previous experience working with explosives).
5. The recent bombing at the Ministry of Minerals and Mines was almost certainly (95-99%) due to anti-Western sentiment in relation to the exploitation of the country's minerals.
6. The Owner and his friend grew up in a village in the south of Kalukistan and attended the polytechnic together. The Owner's friend has been arrested at demonstrations against western presence in Kalukistan. They may share the same attitudes (45-55%) suggesting a possible motive for the Owner.
7. The Owner may have been involved (even odds, 45-55%) in the recent car bomb attack at the Ministry of Minerals and Mines .
8. The strong likelihood of his involvement in bomb-making is sufficient grounds for transfer of the Owner to SOCA for further questioning and processing (Action 4).
9. Detention and interrogation of the Owner could provide further valuable evidence about the bomb-making workshop and the perpetrators of the car-bombing, and would reduce the likelihood of any further attacks involving this site (even odds, 45-55%).
10. His Old Friend, suspect C, may also be implicated in bomb-making as his DNA was present on the soldering iron (even odds, 45-55%), and he too may have a motive, having previously been arrested at an anti-Western demonstration.
11. Active human surveillance and signals surveillance of the Old Friend (Action 3), together with interrogation of the Owner and continuing investigations may provide evidence for establishing the guilt or innocence of the Old Friend and will limit further attacks.
12. There is currently no evidence connecting the Courier or the Customer to the bomb-making operation so they should be released, subject to completion of a full biometric identity file in line with the policy of Special Operations Command Asia (SOCA)'s Identity Intelligence taskforce. This biometric analysis should be completed for all suspects. This Action may be upgraded based on continuing investigation, and interrogation of the Owner.
13. The biometric analysis should be completed for all suspects. This Action may be upgraded based on continuing investigation, and interrogation of the Owner.

Reasoning

Following the explosion of the car bomb outside the Ministry of Minerals and Mines, and following a tip-off, National Gendarmie operatives found a bomb-making workshop in the basement of Oasis Telecom in the Khana Girad district.

Based on the evidence found in the workshop, the NG arrested four suspects: A The Owner, B The Courier, C The Old Friend and D the Customer.

Owner (suspectA) should be subject to Action 4:

1. The Owner (suspect A) claims to have been renting the basement to two foreign workers for the past six months. He claimed that they entered the premises through an external door into the back alley. However he claimed he did not know their surnames or have any transactional records relating to their tenancy.
2. The Owner said that he had not entered the basement during the foreigners' tenancy. This is very unlikely (5-20%) since he could access the basement through a door in his Office, and one would expect the Owner to monitor his
3. property. Furthermore the computer keyboard found in the basement was retired from the Telecom shop four weeks before the raid (testimony of the Courier), making it very likely the Owner had recently entered the basement. The mobile phone found in the basement also came from his shop.
4. The Owner's DNA was found on a soldering iron found in the basement (identified by forensic analysis) indicating his likely involvement (55-80%). However the provenance of the soldering iron is unknown; it may have come from the Telecom premises. The soldering iron also carried the DNA of the Old Friend making it likely (55-80%) his Old Friend had handled the soldering iron. Alternatively, the Old Friend's DNA was inadvertently transferred by the Owner to the soldering iron sometime after dinner, after hand- to-hand contact or via sharing a hookah pipe when they dined together the night before the car bomb incident (unlikely, 20-45%).
5. Using deductive reasoning, the presence of the Old Friend's DNA indicates that almost certainly (95-99%) the Owner either lied when he claimed that he had not entered the basement OR was dishonest in that he had in fact given access to his Old Friend, information he had hidden, and the Old Friend had denied. Either fact implicates the Owner in involvement in the bomb-making workshop. The Owner had verified experience with explosives suggesting he was capable of involvement in bomb-making.
6. On these grounds we recommend detaining the Owner for further interrogation. This action, together with gathering further evidence (see Key Unknowns) should establish his guilt or innocence with respect to the bomb-workshop and to the car-bomb, establish whether there was a connection between the bomb workshop and the car bomb, and also possibly lead to the apprehension of other collaborators.

The Old Friend (suspect C) should be subject to Action 3

1. Given the close friendship between the Owner and the Old Friend (suspect C), their shared background from the provinces, the identification of the Old Friend's DNA on the soldering iron in the bomb-making workshop, and his arrest at an anti-Western demonstration three years previously, it would be prudent to monitor the Old Friend.
2. The Old Friend claimed not to have visited the workshop, and the Courier, who was often at the shop, claims to never have met him, but the Old Friend could have entered the workshop via the door from the rear alley.
3. The possibility of secondary transfer of DNA remains a theoretical possibility (1-20%). It has not been mentioned in the standard DNA methods, and has only been observed in an experimental set-up, when subjects shook hands for 30 seconds before grasping an object. We recommend Action 3 for the Old Friend, including active human surveillance as well as signals surveillance.

The Courier (suspect B) should be subject to Action 1

1. The Courier (Suspect B) is a store attendant and courier at Oasis Telecom. His account appears to be accurate and was corroborated by others and by evidence. He claims no knowledge of the workshop. The fact that his DNA was on the phone and keyboard is not diagnostic since he received the phone from the Customer and he used the keyboard in the shop.
2. Whilst there is no evidence to suggest that he was involved in the bomb-making workshop, it is possible that he was involved as a part of a bomb-making conspiracy. Interrogation of the Owner and the collection of more evidence may be instructive in this regard.
3. In relation to the car bomb, the Courier owns a motorbike which he could have used to transport the car-bomb perpetrators. However he claims to have been at the Store all afternoon and evening.
4. Action 1 is appropriate for the Courier. Should more evidence emerge from ongoing enquiries, from interrogation of the Owner or from signals surveillance of the Old Friend then this response can be upgraded.

The Customer (suspect D) should be subject to Action 1

1. The Customer claims he sold the old phone to the Courier, a fact confirmed by the Courier. The fact that the Customer's DNA was on the phone is therefore not diagnostic with respect to activities within the workshop. It is very unlikely (5-20%) that he was involved in any way.

Further comments regarding the car-bombing

1. With respect to the bombing at the Ministry, the Owner may have been involved since he has no firm alibi. Indeed he admitted he had left the premises to buy lunch and another time 'in the evening, to get some exercise and a coffee', somewhat unconvincing given that his business includes an internet cafe and presumably serves coffee.

2. There is currently no evidence connecting the workshop with the car bomb, but a conjectural scenario presents itself whereby the bomb was made in the workshop, loaded into a car probably the day before the bomb incident, and driven to the Ministry by the Owner some time before 6.30. The Owner could have returned by public transport or by riding pillion on the Courier's motorbike. The bomb could have been detonated by any of the suspects.
3. The most likely motive for the car-bomb was anti-Western sentiments directed toward the delegation discussing access to mineral deposits in the Eastern provinces. If the workshop can be implicated in the car bombing, the Old Friend becomes a stronger person of interest because of his previous arrest at an anti-Western demonstration. However he claims he was caught up in that by mistake.
4. It is possible but not certain that interrogation of the Owner, monitoring of the Old Friend and further investigation at the bomb- making factory might identify those involved in the car bomb attack. There are currently several significant gaps in the evidence (see below)

Key assumptions

We assume that NG operatives did indeed find a bomb-making workshop in the basement of Oasis Telecom. As this information was presented as part of the background briefing, we assumed it to true. Presumably it has been verified by SOCA. However, if SOCA has not verified this report from NG, then our confidence in NG's technical and logical capacity to identify bomb-making operations and their integrity, need to be assessed and factored into all subsequent judgements. That is, a suspect's links to a possible bomb-making workshop would be less justification for higher-level action than a suspect's links to a known bomb-making workshop. We would therefore recommend that this be verified by SOCA.

The National Gendarmerie have not provided us with further evidence confirming that the basement was used as a bomb workshop.

Suspect D is not connected to Telecom Oasis or other suspects except as a customer.

Suspect C is telling the truth about being unable to drive.

Other hypotheses

The Owner is telling the truth, in which case someone else moved, perhaps stole, the computer and mobile phone and placed them in the basement. The Old Friend would then become a strong suspect, now being implicated by his DNA on the soldering iron.

The Owner is telling the truth, and the Courier is lying - the Courier collaborated with the bomb makers and possibly brought equipment, materials and communications (non-telecom) to the bomb makers, using his courier activity as a cover. The Owner was unaware of his subterfuge.. The human intelligence that led the NG to raid the basement was designed to throw the NG off the scent of the true perpetrators of the car bombing (though this conflicts with the Notes we received).

Rogue elements in the NG are feeding SOCA misinformation.

The act of terrorism achieved its aim as the actual targets were the building's workers and not the delegates.

Key unknowns

We have no information on the source of the human intelligence on the bomb workshop, and do not know if it simply identified the presence of the workshop, or whether it connected it in some tangible way to the car-bomb at the Ministry.

We do not know if the Old Friend and the Owner had met together on previous occasions

We know nothing about the provenance of the soldering iron, and whether it had been in the shop.

We have no information on the presence of chemicals or residues, detonators etc within the workshop.

We know nothing about the presence or absence of bomb-related chemical residue on any suspect's clothing,

We know nothing about the possibility of analysis of phone records, and whether the phone (a 'burner') was simply operating with a prepaid phone card, or alternatively, was completely disabled.

We have no information about the car at the Ministry

We know little about who, among the suspects, might have had an old car, or might have been able to purchase a suitable car.

We know nothing about other visitors to the basement who may be suspects. DNA from unknown individuals was found; this could belong to such visitors or it could belong to other shop workers or clients who handled objects prior to their removal from the shop
