# Supplementary figures and images for: Better Together: Reliable Application of the Post-9/11 and Post-Iraq US Intelligence Tradecraft Standards Requires Collective Analysis
Source: Front Psychol. 2019 Jan 7;9:2634. doi: 10.3389/fpsyg.2018.02634 (PMC6330287; doi:10.3389/fpsyg.2018.02634)

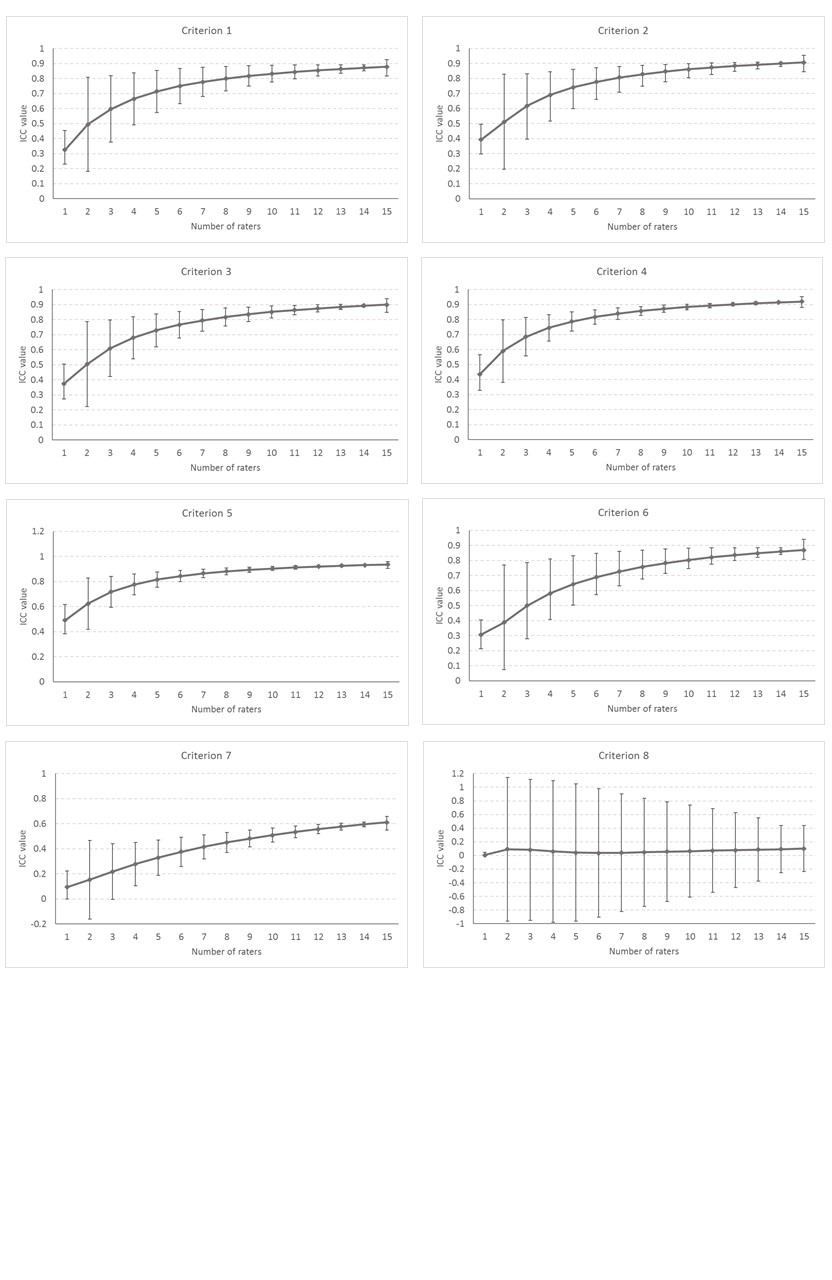

Supplement: Supplementary file 3 [file Image_1.JPEG]
